# No Place Like Home? Disentangling Preferences for HIV Testing Locations and Services Among Men Who Have Sex with Men in China

**DOI:** 10.1007/s10461-018-2366-0

**Published:** 2018-12-18

**Authors:** Stephen W. Pan, Maya Durvasula, Jason J. Ong, Chuncheng Liu, Weiming Tang, Hongyun Fu, Chongyi Wei, Cheng Wang, Fern Terris-Prestholt, Joseph D. Tucker

**Affiliations:** 1 Department of Health and Environmental Sciences, Xi’an Jiaotong-Liverpool University, 111 Ren’ai Road, Suzhou Dushu Lake Higher Education Town, Suzhou, 215123 Jiangsu Province China; 2 UNC-Project China, Guangzhou, China; 3 Department of Economics, Duke University, Durham, NC USA; 4 Faculty of Infectious and Tropical Diseases, London School of Hygiene and Tropical Medicine, London, UK; 5 Central Clinical School, Monash University, Clayton, VIC Australia; 6 Department of Sociology, University of California at San Diego, San Diego, CA USA; 7 School of Medicine, University of North Carolina at Chapel Hill, Chapel Hill, NC USA; 8 Eastern Virginia Medical School, Norfolk, VA USA; 9 School of Public Health, Rutgers University, New Brunswick, NJ USA; 10 Dermatology Hospital, Southern Medical University, Guangzhou, China; 11 Department of Global Health and Development, Faculty of Public Health and Policy, London School of Hygiene and Tropical Medicine, London, UK

**Keywords:** Choice analysis, Stated choice, Stated preference, Gay, Asia, Patient centered

## Abstract

In China, some health departments and gay community-based organizations have begun to offer home-based HIV testing kits in order to augment test uptake among men who have sex with men (MSM). However, HIV test preferences and motivations for home-testing among MSM in China are not well understood. The HIV testing preferences of 803 MSM throughout China were evaluated using single-item assessment and a discrete choice experiment (DCE). In both the single-item assessment and DCE, participants expressed strong preference for free and anonymous testing by health professionals. Both approaches also indicated that naïve testers most prefer home testing. However, among previous testers, the single-item assessment indicated that “home” was the most preferred testing location (vs. hospital or clinic), while the DCE indicated that “home” was the least preferred testing location after controlling for anonymity. HIV home-testing may have limited appeal to previously tested Chinese MSM if anonymity is not maintained.

**Electronic supplementary material:**

The online version of this article (10.1007/s10461-018-2366-0) contains supplementary material, which is available to authorized users.

## Introduction

China has one of the world’s highest reported HIV incidence rates among men who have sex with men (MSM) [1]. Moreover, rates of new infections among MSM in China are rising nationwide, having increased from 3.24 infections/100 person-years (PY) in 2005–2008 to 5.50 infections/100 PY in 2012–2014 [2]. One critical factor contributing to ongoing HIV transmission among MSM is delayed diagnosis of infection [1].

Early HIV diagnosis facilitates earlier initiation of antiretroviral therapy, which in turn ensures better clinical outcomes among people living with HIV and decreases the likelihood of secondary transmissions [3]. However, despite intense efforts to promote routine HIV testing, HIV testing rates among MSM in China remain low. According to a 2015 national online survey, 46% of MSM in China have never received an HIV test [4]. One reason Chinese MSM are not testing more frequently may be because of dissatisfaction with current HIV testing service options [5, 6]. For example, MSM in China have indicated an affinity for testing in public hospitals and clinics [6], but concerns about confidentiality surrounding HIV or MSM status may discourage facility-based testing. Previous studies have qualitatively identified an array of HIV testing service characteristics that Chinese MSM consider when deciding whether to test [6–11], but to date there has been little to no research that has quantitatively examined the *relative* importance of each HIV testing service characteristic. By elucidating how MSM in China weigh the importance of specific HIV testing service attributes, policymakers can better focus HIV testing service optimization on the attributes most likely to increase HIV test uptake.

Discrete choice experiments (DCE) are an established methodology to quantitatively estimate the preferences and relative influence of specific product or service attributes underpinning individual choice decisions [12, 13]. Based on random utility theory, DCEs presume that individuals make rational choices, that is, choices that maximize their satisfaction [14]. DCEs ask individuals to choose between hypothetical product or services options, and then individuals’ stated choices are used to make inferences about their preferences [14]. These stated choices in DCEs enable researchers to quantitatively estimate the conscious or sub-conscious decision-making heuristics and “trade-off” thresholds that individuals use when making discrete choices [15]. Furthermore, by drawing inferences from individuals’ stated choices, DCEs produce estimates of respondent preferences that are less subject to biases introduced when individuals are asked to explicitly report and assess the motivations underlying their choices [15]. Within the health services literature, DCEs have been used to elucidate patient preferences ranging from human papillomavirus vaccination among adolescent females [16] ,to treatment of osteoarthritis among older adults [17], and linkage to HIV care services among the general population [18]. However, few DCEs have examined HIV testing preferences in low or middle income countries (LMIC) [19–23] or among MSM.

In response to this limited understanding of HIV testing preferences among MSM in LMIC, we conducted a DCE to identify potential drivers of HIV testing decisions among MSM in China.

## Methods

### Study Design

From June 2016 to January 2017, the DCE was conducted among MSM by social media throughout China in three stages: (1) Identification of HIV testing service attributes and levels, (2) Generation of the DCE d-efficient design matrix, and (3) Implementation of the DCE.

#### Identification of HIV Testing Service Attributes and Levels

To identify HIV testing service attributes that influence HIV testing decisions among Chinese MSM, we performed a literature review and conducted five focus group discussions each with 4–6 self-identified MSM over 16 years old in Guangzhou, China from June to July 2016 (n = 24). The eligibility criteria for focus group participants was being a person over 16 years old who was born male and has ever had sex with another man. Focus group participants were recruited on social media by a local gay community-based organization and were asked to discuss their decision-making process and service considerations when deciding whether or not to test for HIV [24]. Focus group discussions were moderated by a self-identified gay Chinese MSM, verbally recorded, transcribed, translated from Chinese to English, and analyzed thematically and iteratively [25]. Focus group participants were provided an honorarium of $15 USD.

**Fig. 1:**
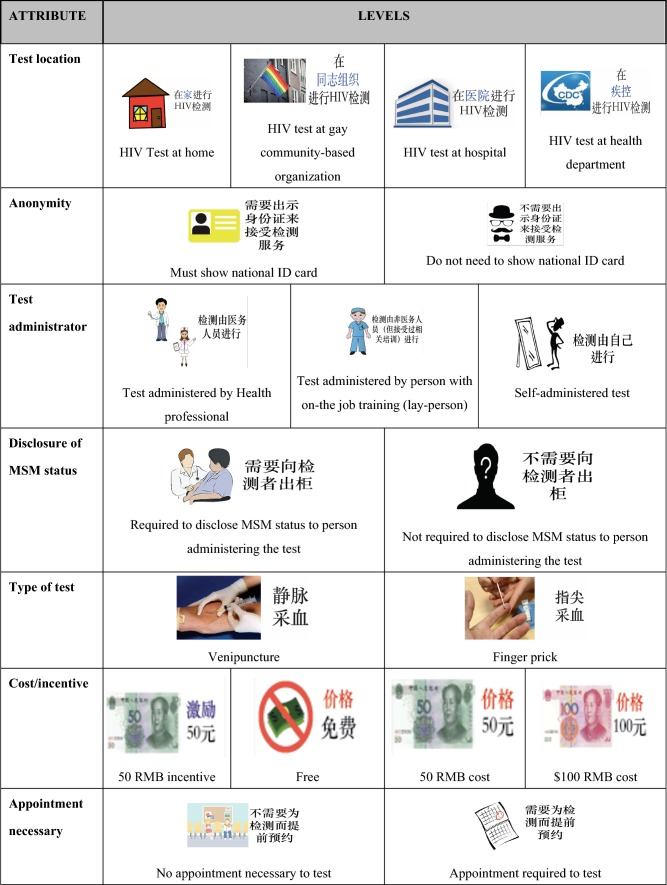
Attributes and levels of the discrete choice experiment

The average age of focus group participants was 26.5 years (StdD: 6.3) and the majority of participants had previously received an HIV test (23/24, 95.8%). Additional sociodemographic characteristics of focus group participants are presented in Supplementary Table S1. Results from focus group discussions revealed seven key HIV testing service attributes and 19 associated levels, which were used to develop DCE choice sets (Fig. 1). Notably, focus group participants consistently expressed that availability of HIV care services to link to was not a consideration when deciding whether or not to test.

Once HIV testing service attributes and levels were identified, we developed pictorial representations of each HIV testing attribute level that would be used in the DCE (e.g., label and picture of a hospital to represent *hospital* as a hypothetical testing location). To ensure that the pictorial representations were clear and intuitive, focus group participants were asked to provide collective feedback on iterations of graphics representing HIV testing attribute levels, and graphics and accompanying text were modified accordingly.

#### Development of the d-Efficient Design Matrix

**Fig. 2:**
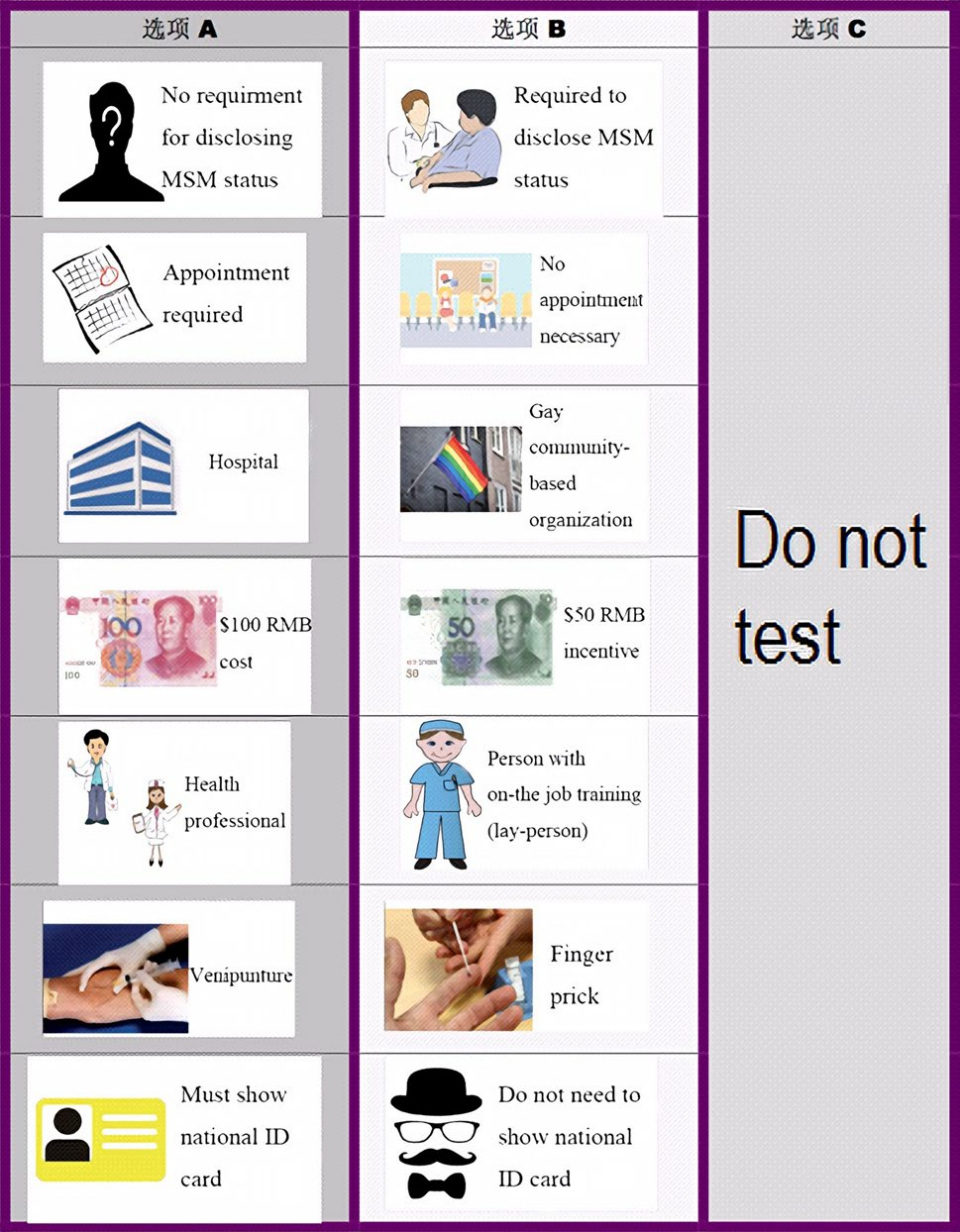
Example of a choice set in the discrete choice experiment

For the current study, we used a d-efficient design matrix [26] that entailed randomizing participants to one of ten blocks and asking each individual to complete six choice tasks (60 unique choice tasks). For each choice task, the participant was instructed to select one of three testing alternatives: testing scenario A, testing scenario B, and opt-out (i.e., do not test) (Fig. 2). The number of blocks and choice tasks presented to each participant was based on considerations of potential survey fatigue and statistical efficiency of each design [27]. Block randomization, choice tasks, randomization of attribute ordering for each choice task, and d-efficiency statistics were produced in NGENE (ChoiceMetrics, 2014). No implausible testing scenarios were included (e.g., being required to disclose one’s true name to the test administrators if *self* was the test administrator). In order to improve the precision of parameter estimates, the d-efficient design produced in NGENE used parameter estimate priors generated from an online pilot test conducted among 96 self-identified MSM from two Chinese provinces.

#### Implementation of the DCE

The DCE was conducted online from January 8–31, 2017. Participants were recruited via multiple gay community based organizations and gay social networking portals throughout China. To participate in the study, individuals were required to self-report having been born biologically male, being over 16 years old (the legal age of adult consent), having ever had anal or oral sex with another man, and never having been diagnosed with HIV. To ensure ability to adequately assess preferences among subgroups of Chinese MSM, the following 1:1 ratio sampling quotas were imposed: income above and below $450 USD per month, education above and below high school, and history of MSM behavior disclosure to healthcare worker (yes/no). Sub-analysis indicated that propensity to select the “opt-out” alternative did not significantly vary by education, income, or MSM disclosure status. Participants in the DCE pilot test survey received a $7.50 USD phone credit honorarium delivered to their cell phone.

### Statistical Analyses

Data analysis was conducted using three successive models: Multinomial logit (MNL), Mixed logit (MXL), and mixed logit with interaction terms (MXL-I). The MNL model included only main effects that provided overall averages of the study sample’s HIV testing preferences. However, because the MNL approach does not account for unobserved preference heterogeneity between individuals or repeated observations collected from a single individual [28], we extended our analysis to include a MXL model. The MXL model only included main effects, all of which were set as random, assumed to be normally distributed, and modeled as non-linear, nominal categorical variables. Lastly, we used an MXL-I model that included parameter estimates for both attribute levels and interactions. In order to assess potential differences in testing preferences, the MXL-I model included two-way interaction terms between all design attributes and HIV testing history (ever received HIV test, yes vs. no). Model fit for the MXL and MXL-I models were compared using the log-likelihood ratio test. All analyses were conducted using the MLOGIT package in R and 1000 Halton draws. Effects and indicator coding were used for the design attributes and individual-specific characteristics, respectively. To facilitate clearer interpretation of relative satisfaction, design attribute coefficients were scaled from 0 to 10.

The relative importance of each study attribute was calculated by dividing the range of parameter estimates for a given attribute, by the sum of parameter estimate ranges for all attributes [28]. Essentially, the relative importance for each attribute represents the proportion of DCE stated choices that were determined by the given attribute, excluding the influence of the opt-out selection and unmeasured factors (i.e., error terms). Confidence intervals for estimates of attribute relative importance were derived by calculating the standard error of each attribute’s range using the following formula:

$$ \surd [\left( {variance\,of\,attribute\,level\,A} \right) + \left( {variance\,of\,attribute\,level\,B} \right) + \left( {2*covariance\,between\,attribute\,levels\,A\,and\,B} \right)] $$

[28]. Stratification was used to calculate the relative importance of testing attributes by HIV testing history (ever tested vs. never tested). Relative importance estimates and confidence intervals were based on MXL models.

### Ethical Review

Study protocols were approved by the institutional review boards of the University of North Carolina at Chapel Hill (Study number 16-1860) and the Guangdong Provincial Dermatology Hospital. Each study participant was presented with an online consent form and provided informed consent prior to enrollment.

## Results

In 23 days, the DCE survey link was clicked 3319 times. 2505 respondents failed to meet the study eligibility criteria or quit before eligibility could be established, and 11 respondents were removed after deduplication of identical phone numbers. The 803 eligible participants completed 4738 choice tasks. Alternative “A” (choice on the left side) and alternative “B” (choice on the right side) had comparable probabilities of being selected (47% vs. 45%, respectively); the “opt-out” alternative was selected in 8% of all choice tasks.

### Participant Characteristics

Participant characteristics are presented in Table 1. Overall, study participants were relatively young (median age: 24 years old), mostly single (86%), mostly self-identified as gay (78%), and most had previously tested for HIV (68%). Distribution of education, income, and disclosure of MSM identity to health care providers aligned closely to the quota sampling scheme. Participants who never received an HIV test were more likely to be less than 28 years old (79% vs. 69%), have only elementary or middle school education (21% vs. 15%), and have income less than 217 USD per month (28% vs. 19%) compared to men who had ever received an HIV test.

**Table 1:** Participant sociodemographics among MSM in China (n = 803)

	Code	Total n (%)	Among those who ever received an HIV test (%)	Among those who never received an HIV test (%)	p value
Age (years)					
1^st^ quantile < 21	1	187 (23)	(18)	(34)	< 0.0001
2^nd^ quantile 21–23	2	191 (24)	(25)	(21)	
3^rd^ quantile 24–28	3	199 (25)	(25)	(24)	
4^th^ quantile > 28	4	226 (28)	(31)	(21)	
Educational attainment					
Elementary/middle	1	137 (17)	(15)	(21)	0.02
High school	2	287 (36)	(36)	(36)	
Vocational college	3	129 (16)	(15)	(19)	
Four-year college and above	4	250 (31)	(34)	(25)	
Urban residency status					
Official urban resident	1	421 (52)	(55)	(48)	0.08
Rural resident	0	382 (48)	(45)	(52)	
Current marital status					
Single	1	689 (86)	(84)	(90)	< 0.01
Married	2	77 (10)	(10)	(9)	
Separated/divorced/widowed	3	37 (5)	(6)	(1)	
Sexual orientation					
Gay	1	623 (78)	(80)	(73)	0.10
Heterosexual	0	144 (18)	(16)	(23)	
Bisexual	0	8 (1)	(1)	(1)	
Unsure	0	28 (3)	(4)	(3)	
Income, USD/month					
< 217	1	177 (22)	(19)	(28)	< 0.01
217–433	2	246 (31)	(31)	(30)	
434–724	3	235 (29)	(29)	(30)	
725–1159	4	89 (11)	(13)	(8)	
> 1159	4	56 (7)	(8)	(4)	
n		803	550	253	

### Single-Item Assessment of Testing Preferences

Single-item assessment of testing preferences (i.e., participants reporting their HIV testing preferences independently for each attribute) are presented in Table 2. Regarding test location, participants most preferred to test at home (34%), followed by testing at gay community-based organizations (25%), and local health departments (18%). The most popular pricing models were free testing (55%) and incentivized testing (24%). Two out of three participants preferred walk-in testing (66%), while only one in five preferred appointment-based testing. The majority of individuals preferred to test anonymously (75%), not be required to disclose their same-sex sexual activities (60%), and to be tested by a trained health professional (60%). Finger-prick testing was preferred over venous blood testing (48% vs. 27%), but one in four participants was indifferent to the test type (25%). Participants who had never tested before had a greater preference for testing at home (45% vs. 29%), self-testing (33% vs. 15%), finger prick testing (54% vs. 46%), and real-name testing (22% vs. 14%).

**Table 2:** Single-item assessment of participants’ HIV testing preferences among MSM in China (n = 803)

	Total n (%)	Among those who ever received an HIV test (%)	Among those who never received an HIV test (%)	p value
Test location^a^				
Home	271 (34%)	(29)	(45)	< 0.0001
Gay community-based organization	201 (25%)	(26)	(23)	
Hospital	75 (9%)	(10)	(8)	
Local health department	144 (18%)	(21)	(11)	
Indifferent	109 (14%)	(14)	(13)	
Cost/incentive of test^a^				
$7.50 USD incentive	194 (24%)	(28)	(16)	< 0.001
Free	438 (55%)	(53)	(58)	
Pay $ 7.50 USD	34 (4%)	(3)	(6)	
Pay $15 USD	44 (6%)	(6)	(4)	
Indifferent	90 (11%)	(9)	(15)	
Appointment versus walk-in testing^a^				
Walk-in	532 (66%)	(67)	(64)	0.12
Appointment necessary	159 (20%)	(18)	(24)	
Indifferent	112 (14%)	(15)	(12)	
Disclosure of same-sex sexual activities to test administrator^a^				
Not required to disclose	483 (60%)	(60)	(60)	0.27
Required to disclose	189 (24%)	(22)	(26)	
Indifferent	129 (16%)	(17)	(13)	
Test administrator^a^				
Trained health professional	484 (60%)	(64)	(52)	< 0.001
Individual with on the job training to administer test	89 (11%)	(12)	(9)	
Self-test	164 (20%)	(15)	(33)	
Indifferent	65 (8%)	(9)	(6)	
Test type^a^				
Finger prick	387 (48%)	(46)	(54)	< 0.001
Venous	217 (27%)	(31)	(18)	
Indifferent	198 (25%)	(23)	(28)	
Anonymity^a^				
Not required to show national ID card	604 (75%)	(79)	(67)	< 0.01
Required to show national ID card	132 (16%)	(14)	(22)	
Indifferent	66 (8%)	(7)	(11)	
n	803	550	253	

^a^Single response option

### Overall Design Attribute Effects

#### MNL Model

Supplementary Table S2 shows participants’ attribute-specific HIV testing preferences based on results of the MNL analysis. In contrast to the single-item assessment, home was the least preferred testing location (β = − 0.10, p < 0.01). Notably, participants expressed slightly stronger preference for free testing over testing with monetary incentives (β = 0.32 vs. β = 0.23). Preference ranking for all other parameter estimates were in the order as expected (e.g., stronger preference for cost of $7.50 USD test vs. $15 USD test).

#### MXL Model

Table 3 shows results of the MXL analysis. Each attribute contained at least one level with statistically significant standard deviation estimates of the coefficient, thus implying substantial heterogeneity of preference weights across respondents for all attributes [28].

**Table 3:** HIV testing preferences of MSM in China (MXL model)

Design attributes and levels	Coefficient	SE	StdD	SE
Test location				
Home	− 0.14*	0.05	0.75***	0.11
Community-based organization	0.04	0.05	0.59***	0.12
Hospital	− 0.05	0.05	− 0.01	0.65
Health department	0.15**	0.05	0.01	0.90
Identifier collected at test time				
Must show ID card	− 0.33***	0.04	0.58***	0.07
Do not need to show ID card	0.33***	0.04	0.61***	0.07
Test administrator				
Self-test	− 0.18	0.19	0.98***	0.12
Person with on-the job training	− 0.24*	0.10	− 0.25*	0.12
Health professional	0.41***	0.11	0.10	0.31
MSM identity disclosure				
Required to disclose MSM activity	− 0.13***	0.03	0.46***	0.07
NOT required to disclose MSM activity	0.13***	0.03	0.43***	0.07
Type of test				
Venipuncture	0.06	0.03	0.38***	0.08
Finger prick	− 0.06	0.03	0.31***	0.09
Cost/incentive				
7.50 USD incentive	0.31***	0.05	0.40**	0.14
Free	0.49***	0.06	0.66***	0.12
7.50 USD cost	− 0.22***	0.06	− 0.04	0.47
15 USD cost	− 0.59***	0.06	0.01	0.52
Scheduling				
Walk-in	0.06	0.03	0.14	0.17
Appointment necessary	− 0.06	0.03	0.22*	0.11
Nonrandom parameter				
Opt-out ASC	− 0.89***	0.05		
Model fit statistics				
Number of individuals	803			
Number of completed choice sets	4738			
Log-likelihood function	− 4075.5			

*p < 0.05; **p < 0.01; ***p < 0.001

*StdD* Standard deviation, *SE* standard error, *ASC* alternative-specific constant

#### MXL-I Model

Table 4 shows results of the MXL-I analysis. The MXL-I model extends the MXL model to explore preference heterogeneity by testing experience. The log-likelihood ratio test indicated that the MXL-I model fit significantly better than the MXL model (p < 0.001, χ^2^ = 42.3, 13 DF). The MXL-I analysis indicated that preference for testing at home was significantly stronger among test-naïve men, compared to previous testers (β = − 0.58, p < 0.001). Participants with testing experience expressed significantly stronger preference for testing at the health department (β = 0.41, p < 0.001). In addition, naïve testers were significantly more likely to choose the opt-out choice, compared to men with testing experience (β = 0.23, p < 0.05).

A sensitivity analysis was conducted to examine how sociodemographic may have influenced preferences of naïve and experienced testers. The sensitivity analysis entailed re-running the MXL-I analysis among sub-samples stratified by age, sexual orientation, and income. Results of the sensitivity analysis indicated that naïve testers’ stronger preference for home testing and weaker preference for testing at the health department (compared to experienced testers) was consistent within each stratum of age, sexual orientation, and income levels (results available upon request).

### Scaled HIV Testing Preferences

**Fig. 3:**
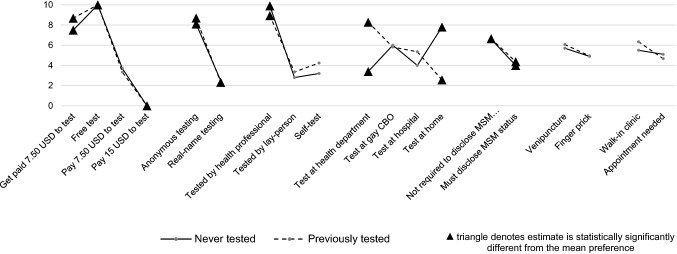
Scaled estimates of HIV testing preferences among MSM in China, by testing history (MXL-I model) (n = 803)

Figure 3 illustrates scaled HIV testing preferences, enabling direct comparisons between testing levels and attributes. Larger values indicate stronger preference for a specific testing characteristic. Results showed that switching from real-name testing to anonymous testing was as influential on participants’ stated preferences as changing from $7.50 USD out-of-pocket testing to free testing, or changing the test administrator from a lay-person to a health professional (gain of approximately 6 points on rescaled scale).

**Table 4:** HIV testing preferences of MSM in China (MXL-I model)

Design attributes and levels	Coefficient	SE	StdD	SE
Test location				
Home	0.25**	0.10	0.72***	0.11
Gay community-based organization (CBO)	0.07	0.10	0.58***	0.12
Hospital	− 0.13	0.09	0.02	0.77
Health department	− 0.19*	0.09		
Identifier collected at test time				
Must show ID card	− 0.28***	0.06	0.57***	0.07
Do not need to show ID card	0.28***	0.06		
Test administrator				
Self-test	− 0.21	0.33	0.97***	0.12
Person with on the job training	− 0.25	0.17	− 0.24*	0.12
Health professional	0.46*	0.18		
MSM identity disclosure				
Required to disclose MSM activity	− 0.13*	0.05	0.47***	0.07
NOT required to disclose MSM activity	0.13*	0.05		
Type of test				
Venipuncture	0.04	0.06	0.37***	0.08
Finger prick	− 0.04	0.06		
Cost/incentive				
7.50 USD incentive	0.22*	0.09	0.39**	0.15
Free test	0.47***	0.11	0.66***	0.12
7.50 USD cost	− 0.16	0.09	− 0.02	0.50
15 USD cost	− 0.53***	0.09		
Scheduling				
Appointment necessary	− 0.02	0.06	0.12	0.19
Walk-in	0.02	0.06		
Nonrandom parameter				
Opt-out ASC	− 0.74***	0.09		
Interaction terms				
Ever had HIV test * Home	− 0.58***	0.12		
Ever had HIV test * Gay CBO	− 0.03	0.12		
Ever had HIV test * Hospital	0.12	0.11		
Ever had HIV test * Must show ID card	− 0.07	0.08		
Ever had HIV test * Self-test	0.07	0.40		
Ever had HIV test * Person with on the job training	0.00	0.21		
Ever had HIV test * Required to disclose MSM activity	0.00	0.07		
Ever had HIV test * Venipuncture	0.02	0.07		
Ever had HIV test * 7.50 USD incentive	0.14	0.11		
Ever had HIV test * Free test	0.04	0.13		
Ever had HIV test * 7.50 USD cost	− 0.08	0.12		
Ever had HIV test * Appointment necessary	0.11	0.07		
Ever had HIV test * optout	− 0.23*	0.11		
Model fit statistics				
Number of individuals	803			
Number of observations	4738			
Log-likelihood function	− 4054.4			

*p < 0.05; **p < 0.01; ***p < 0.001

*StdD* Standard deviation, *SE* standard error, *ASC* alternative-specific constant

### Relative Importance

Table 5 presents the relative importance of HIV testing attributes as percentages, stratified by HIV testing history. Overall, cost/incentive was the most important attribute (34.0%, 95% CI 31.2–36.7%), followed by anonymity (20.8%, 95% CI 18.1–23.3%), and test administrator (20.4%, 95% CI 7.0–30.4%). Testing location was of modest importance (9.2%, 95% CI 5.7–12.4%), but disclosure of MSM activity (7.9%, 95% CI 5.3–10.4%), test type (3.9%, 95% CI 1.0–6.7%), and appointment scheduling (3.7%, 95% CI 1.0–6.2%) were of limited importance. The relative importance of attributes was generally similar between participants with different testing histories.

**Table 5:** Relative importance of HIV testing attributes among MSM in China (MXL models) (n = 803)

	Location	Anonymity	Test administrator	Disclosure of MSM activity	Test type	Cost/incentive	Appointment/walk-in
Overall	9.2% (5.7–12.4%)	20.8% (18.1–23.3%)	20.4% (7.0–30.4%)	7.9% (5.3–10.4%)	3.9% (1.0–6.7%)	34.0% (31.2–36.7%)	3.7% (1.0–6.2%)
Ever tested	17.8% (14.3–21.0%)	19.3% (16.3–22.0%)	17.0% (3.1–27.5%)	6.7% (3.9–9.3%)	3.6% (0.4–6.5%)	30.8% (27.3–33.8%)	5.0% (2.2–7.7%)
Never tested	13.9% (8.0–19.2%)	18.5% (14.2–22.4%)	22.1% (15.3–27.9%)	7.7% (3.5–11.5%)	2.6% (2.2–6.9%)	33.5% (28.0–38.3%)	1.6% (0.0–5.7%)

## Discussion

Today, MSM in China have increasingly diverse HIV testing options. Health professionals at hospitals and local health departments often provide free HIV screening tests for self-identified MSM [29], though such testing conditions may not be appealing to MSM reluctant to disclose their MSM status to heterosexual individuals for fear of stigmatization and/or discrimination [30]. Larger cities often offer free and anonymous testing at gay community-based organizations staffed by MSM, but qualitative research suggests that some MSM may prefer testing at specialized hospitals staffed by formally trained health professionals [31]. Most recently, online sales of HIV self-testing kits now enable individuals to conduct self-administered home-based testing, an approach which has received monetary and explicit policy support from the Chinese government and local health departments [32].

However, in order to effectively deploy multi-pronged testing strategies that will increase test uptake, it is imperative to understand the component drivers of HIV testing preferences. Novel findings from this study are instructive for programs seeking to augment HIV testing among MSM in China, particularly government programs that may include promotion of home-based HIV testing kits. This study extends the literature of HIV testing preferences by using an experimental design to disentangle the motivations underpinning location and service preferences for HIV testing.

The DCE revealed several novel findings which had not been identified in previous HIV testing preference studies among Chinese MSM [6–11]. First, results indicated that MSM with different HIV testing histories have significantly different preferences for where to test. Among test-naïve men, home and the health department were the most and least favored testing locations, respectively. However, the exact opposite was true among men who had previously tested. One possible reason test-naïve men preferred to test at home is because they were expressing stated preferences for their hypothetical first HIV test, which may be perceived differently than subsequent tests. That is, men without any personal testing experience may feel more intimidated or apprehensive about testing at government health departments [6], and hence prefer to test in the comfort of their own home. Men with prior testing experience were likely more familiar with testing procedures, and therefore may have had fewer unanswered questions or concerns about testing at a health department. Alternatively, it is possible that naïve testers’ preference for home-testing was directly contributing to their lack of testing experience. That is, the absence of ideal home testing options may have been a key contributing factor for why naïve testers had not yet tested.

Second, results from the DCE illuminated the potential limitations of measuring preferences by single-item assessment alone. According to the single-item assessment, home-testing was the most preferred testing location among previous testers. However, after accounting for factors such as anonymity and disclosure of MSM status, findings from the DCE indicated that home testing was the *least* preferred location among previous testers. This discrepancy between the single-item assessment and DCE results suggests that the popularity of home testing among previous testers was not due to any inherent qualities of the home environment, but rather because anonymity and confidentiality were relatively more assured by home testing. Future preference studies should be cautious about what types of conclusions can be inferred from single-item assessment methods.

Third, study results are among the first to rank the weighted importance of HIV testing attributes among MSM. Previous research has used qualitative data or single-item assessment to enumerate influential HIV testing service/product considerations among Chinese MSM [6–11], but methodological limitations of such study designs make it difficult to estimate measures of relative importance independently, which can lead to incorrect inferences. Using an experimental design that enabled estimates of testing attribute relative importance, we were able to identify which HIV testing service/product attributes exerted the strongest influence on stated preference, and potentially HIV test uptake. Specifically, cost, anonymity, and test administrator were the three most important testing attributes, collectively influencing ~ 75% of all stated preferences. These findings are partially consistent with stated preference studies conducted in Africa [20, 22] and the United States [33], which also indicated cost as the most important attribute for patient HIV testing preferences.

However, a previous HIV testing stated preference study among MSM in the US did not identify anonymity as an especially important attribute [33]. This discrepancy may be partly explained by relatively stronger social stigmatization of homosexuality and same-sex sexual behaviors in China compared to the US [34]. Although same-sex sexual behaviors are legal and many sexual minority advocacy organizations operate openly [34], public expressions of sexual minority issues remain potentially subject to censure and censorship [35]. Given the social costs of disclosing MSM behaviors and identities in China, current government efforts to standardize mandatory real-name HIV testing [36] will almost certainly discourage HIV test uptake among MSM throughout China.

This study has several implications for HIV testing policy and research. First, findings indicate that home-based HIV self-test kits may have limited appeal to Chinese MSM with previous testing experience if anonymity is not maintained. It is now possible for individuals to purchase HIV self-test kits from the internet in China, but a substantial proportion of MSM may lack ability to routinely purchase and administer their own self-tests. As noted in this study and previous research from the US [33], cost is the single most important testing attribute for MSM when considering testing options. As government public health organizations in China begin to promote HIV self-testing kits to MSM alongside conventional facility-based testing services [37], it is incumbent for relevant policy makers to bear in mind that the appeal of home-based testing will be severely diminished if real-name testing is required. Simultaneously offering anonymous home-based and facility-based testing options is likely to satisfy the preferences of naïve and previous testers alike.

Second, study findings suggest that small monetary incentives are unlikely to significantly increase test uptake among Chinese MSM, when compared to free testing. In both the single-item assessment and the DCE, participants expressed stronger preference for free testing, rather than testing with monetary incentives. From a classical economic perspective, this preference for free testing over incentivized testing may appear irrational and unexpected. However, this finding accords well with robust research from behavioral economics [38], which suggests that introducing monetary incentives can actually diminish the intrinsic motivation of getting tested for HIV (e.g., sense of satisfaction derived from taking care of one’s health). Another possible explanation is that MSM may be distrustful of monetary incentives used to promote HIV testing. Campaigns and programs to boost HIV testing among MSM in China should consider non-monetary incentives, or approaches that can enhance intrinsic motivations.

### Limitations

Several study limitations should be noted. First, generalizability may be limited because the HIV testing service attributes and levels used in the DCE were based on focus groups from a single, urban city in Southern China. It is possible that MSM in rural or Northern China may have qualitatively different HIV testing service considerations when deciding whether to test. Second, study findings may not be generalizable to completely off-line MSM in China, although by using sampling quotas, we were able to ensure greater sociodemographic representation. Third, measures of relative importance only reflect observed variation, and do not take into account the unobserved variation in stated preferences. Fourth, the stated preferences of HIV testing services may be distinct from HIV testing decisions in the real-world, and inferences about HIV testing decisions in the real-world must be made judiciously [12].

## Conclusion

Supplementing conventional clinic-based testing with home-based HIV self-testing kits is a promising approach to increase HIV test uptake among MSM in China. However, findings from this study suggest that providing home-test kits to Chinese MSM may have limited impact on routine HIV test uptake unless testing is anonymous. Future studies should consider applying DCEs as a means of disentangling the motivations underpinning patient preferences at other stages of the HIV cascade of care.

## Electronic supplementary material

Below is the link to the electronic supplementary material.

Supplementary material 1 (DOCX 16 kb)
