# Supplementary material for: No Place Like Home? Disentangling Preferences for HIV Testing Locations and Services Among Men Who Have Sex with Men in China
Source: AIDS Behav. 2018 Dec 18;23(4):847–59. doi: 10.1007/s10461-018-2366-0 (PMC6458980; doi:10.1007/s10461-018-2366-0)
Supplement: Supplementary file 1 — Supplementary material 1 (DOCX 16 kb) [file 10461_2018_2366_MOESM1_ESM.docx]

**APPENDICES**

**Supplementary Table SI: Sociodemographic characteristics of focus group participants**

|  | n (%) |
| --- | --- |
| Age (mean, SD) | 26.5, 6.3 |
| Education attainment |  |
| Elementary/Middle | 0 (0.0) |
| High school | 4 (16.7) |
| Post-secondary education | 7 (29.2) |
| Four-year college and above | 13 (54.1) |
| Urban residency status |  |
| Official urban resident | 13 (54.2) |
| Rural resident | 11 (45.8) |
| Marital status |  |
| Never married | 24 (100.0) |
| Married | 0 |
| Separated/divorced/widowed | 0 |
| Income, USD/month |  |
| <217 | 2 (8.3) |
| 217-433 | 2 (8.33) |
| 434-724 | 12 (50.0) |
| 725-1159 | 5 (20.83) |
| >1159 | 3 (12.5) |
| Ever tested for HIV |  |
| Yes | 23 (95.8) |
| No | 1 (4.2) |

| **Supplementary Table SII: Multinomial logit model of MSM HIV testing preferences in China** | | |
| --- | --- | --- |
|  | Coefficient | SE |
| Design attributes and levels |  |  |
| Test location |  |  |
| Home | -0.10** | 0.04 |
| Community-based organization | 0.02 | 0.04 |
| Hospital | -0.03 | 0.04 |
| Health department | 0.11** | 0.04 |
| Identifier collected at test time |  |  |
| Must show ID card | -0.23*** | 0.02 |
| Do not need to show ID card | 0.23*** | 0.02 |
| Test administrator |  |  |
| Self-test | -0.07 | 0.12 |
| Person with on-the job training | -0.17** | 0.06 |
| Health professional | 0.24*** | 0.07 |
| MSM identity disclosure |  |  |
| Required to disclose MSM activity | -0.08*** | 0.02 |
| NOT required to disclose MSM activity | 0.08*** | 0.02 |
| Type of test |  |  |
| Venipuncture | 0.04 | 0.02 |
| Finger prick | -0.04 | 0.02 |
| Cost/incentive |  |  |
| 7.50 USD incentive | 0.23*** | 0.04 |
| Free | 0.32*** | 0.04 |
| 7.50 USD cost | -0.16*** | 0.04 |
| 15 USD cost | -0.40*** | 0.04 |
| Scheduling |  |  |
| Walk-in | 0.06** | 0.02 |
| Appointment necessary | -0.06** | 0.02 |
|  |  |  |
| Nonrandom parameter |  |  |
| Opt-out ASC | -0.86*** |  |
|  |  |  |
| Model fit statistics |  |  |
| Number of individuals | 803 |  |
| Number of completed choice sets | 4476 |  |
| Log-likelihood function | -4194.8 |  |
